# Establishment of a neutrophil extracellular trap-related prognostic signature for colorectal cancer liver metastasis and expression validation of CYP4F3

**DOI:** 10.1007/s10238-024-01378-0

**Published:** 2024-05-25

**Authors:** Xiao-Li Tang, Zi-Yang Xu, Jiao Guan, Jing Yao, Xiao-Long Tang, Zun-Qiang Zhou, Zheng-Yun Zhang

**Affiliations:** 1 Department of Surgery, Shanghai Sixth People’s Hospital Affiliated to Shanghai Jiao Tong University School of Medicine, 600 Yishan Road, Shanghai, 200233 China; 2 Department of General Surgery, Shanghai Eighth People’s Hospital, 8 Caobao Road, Shanghai, 200235 China

**Keywords:** Neutrophil extracellular trap, CYP4F3, Colorectal cancer, Liver metastasis

## Abstract

Liver metastasis stands as the primary contributor to mortality among patients diagnosed with colorectal cancer (CRC). Neutrophil extracellular traps (NETs) emerge as pivotal players in the progression and metastasis of cancer, showcasing promise as prognostic biomarkers. Our objective is to formulate a predictive model grounded in genes associated with neutrophil extracellular traps and identify novel therapeutic targets for combating CRLM. We sourced gene expression profiles from the Gene Expression Omnibus (GEO) database. Neutrophil extracellular trap-related gene set was obtained from relevant literature and cross-referenced with the GEO datasets. Differentially expressed genes (DEGs) were identified through screening via the least absolute shrinkage and selection operator regression and random forest modeling, leading to the establishment of a nomogram and subtype analysis. Subsequently, a thorough analysis of the characteristic gene CYP4F3 was undertaken, and our findings were corroborated through immunohistochemical staining. We identified seven DEGs (ATG7, CTSG, CYP4F3, F3, IL1B, PDE4B, and TNF) and established nomograms for the occurrence and prognosis of CRLM. CYP4F3 is highly expressed in CRC and colorectal liver metastasis (CRLM), exhibiting a negative correlation with CRLM prognosis. It may serve as a potential therapeutic target for CRLM. A novel prognostic signature related to NETs has been developed, with CYP4F3 identified as a risk factor and potential target for CRLM.

**Supplementary Information:**

The online version contains supplementary material available at 10.1007/s10238-024-01378-0.

## Introduction

Colorectal cancer (CRC) ranks as the second and third most common cancer among women and men worldwide, respectively, contributing to nearly 900,000 annual fatalities [1]. The primary cause of mortality in CRC patients is metastasis, with approximately 20% presenting metastatic disease at the time of diagnosis, predominantly in the liver [2, 3]. Therefore, comprehending the molecular mechanisms underlying colorectal cancer liver metastasis (CRLM) and developing effective diagnostic methods for liver metastasis in CRC patients are crucial, constituting the central focus of numerous research endeavors.

Neutrophils, integral to immune defense, have been the subject of extensive research in recent years, particularly regarding the formation and function of neutrophil extracellular traps (NETs) [4, 5]. NETs, web-like structures comprising DNA-histone complexes and proteins released by activated neutrophils, exhibit both antitumoral and pro-tumoral effects [6, 7]. They typically form via two pathways: one involving cell death known as NETosis and the other being a non-lytic form of NETosis that operates independently of cell death [4]. The role of NETs in cancer seems to contingent on the immune system’s status and its interaction with the tumor microenvironment. Evidence suggests that NETs contribute to the progression and metastasis of various cancers [6, 8, 9]. Deng et al. highlighted that the activation of the receptor tyrosine kinase discoid domain receptor 1 (DDR1) in pancreatic ductal adenocarcinoma (PDCA) cells stimulates NETs formation and tumor invasion [10]. Khan et al. delineated several mechanisms through which the CRC microenvironment induces NETs formation and how NETs foster the proliferation and invasion of CRC cells [11]. Nevertheless, CRLM is a multifaceted process involving numerous factors and pathways, and research gaps remain to be addressed. CYP4F3 is one of the NETs-related genes (NETRGs). As a member of the cytochrome P450 (CYP) family, it encodes two unique enzymes, CYP4F3A and CYP4F3B, which facilitate the resolution of inflammatory responses by degrading leukotrienes, arachidonic acid, and prostaglandins [12–14]. Emerging research suggests a significant role for CYP4F3 in oncology [15–18].

Within the scope of our study, we obtained six gene expression profiles of CRC patients from the Gene Expression Omnibus (GEO) database to comprehensively assess the correlation between NETRGs and the progression of CRLM, as well as to conduct subsequent evaluations. We devised a nomogram with the capacity to predict the likelihood of CRLM occurrence, and a separate nomogram capable of forecasting the survival duration of individuals with CRLM specially. Furthermore, we examined variations in CYP4F3 across distinct CRLM subtypes, aiming to elucidate the potential molecular mechanisms, particularly from the standpoint of immune infiltration. Our findings may contribute significantly to the advancement of innovative predictive methods for CRLM and the identification of therapeutic biomarkers.

## Materials and methods

### Data processing and NETRGs expression levels acquisition

**Fig. 1:**
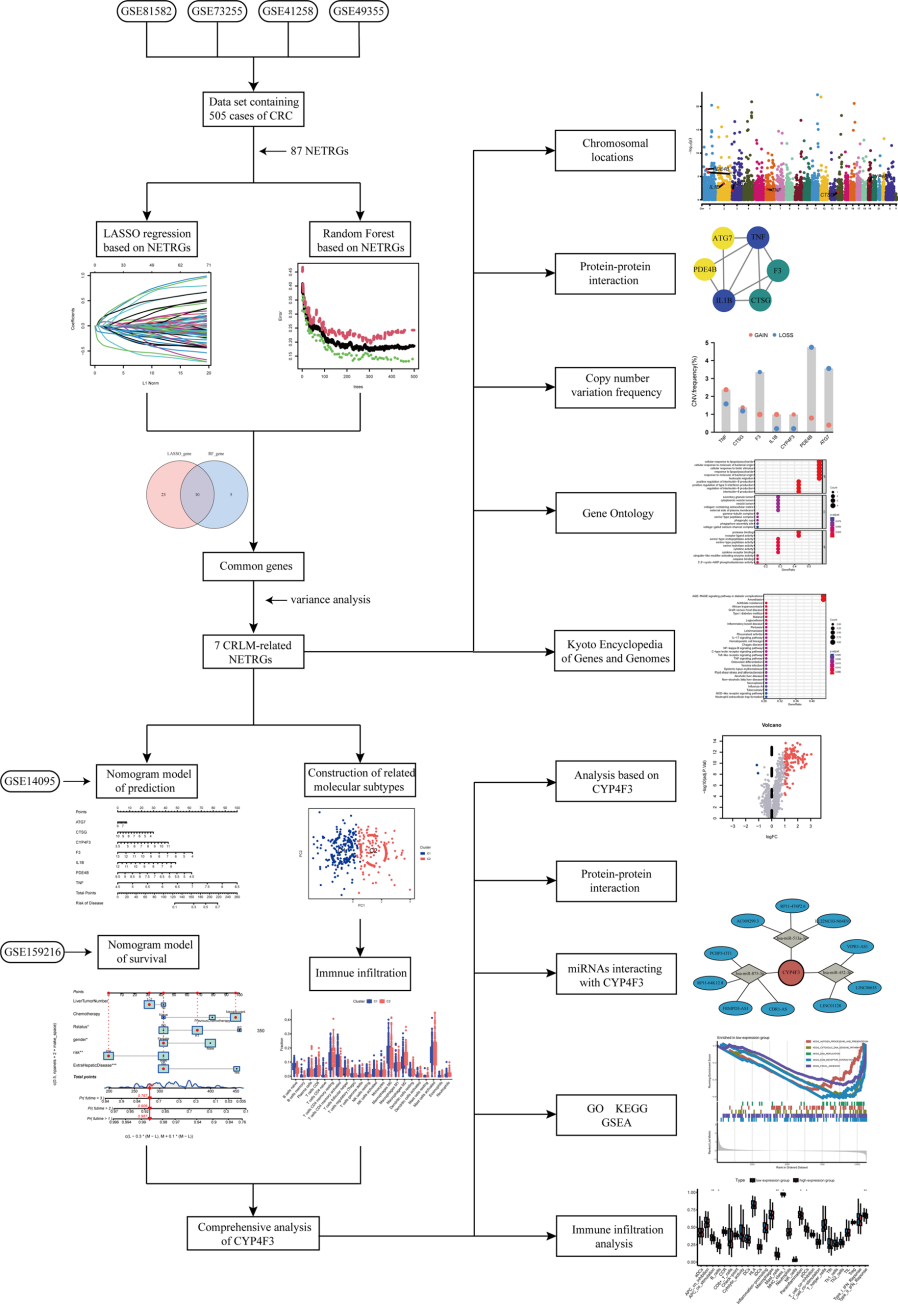
The illustrated schematic representation of the comprehensive analytical process

We retrieved the RNA-seq of GSE81582, GSE73255, GSE41258, GSE49355, GSE14095, and GSE159216 from the GEO database (https://www.ncbi.nlm.nih.gov/geo/). These samples underwent annotation using probe information, normalization, and batch effect correction. All data processing was executed using R software version 4.3.0. The “normalizeBetweenArrays” function of the package “limma” (3.56.2) was employed to obtain normalized data [19]. The first four datasets were merged using the package “sva” (3.48.0), and batch effects were mitigated using the “removeBatchEffect” function of “limma.” Each sample was tagged as “Control” or “Treat” to differentiate between primary colorectal cancer (pCRC) and CRLM samples. Subsequently, we procured the NETRGs from pertinent literature and extracted the expression levels of them [20–22]. The illustrated schematic representation of the comprehensive analytical process is depicted in Fig. 1.

### Establishment of LASSO, RF, and acquisition of DEGs

We utilized 505 samples (235 pCRC samples and 270 CRLM samples) from the four datasets (GSE81582, GSE73255, GSE41258, and GSE49355) to establish the least absolute shrinkage and selection operator (LASSO) regression and random forest (RF) models to identify NETRGs associated with CRLM. A differential analysis was conducted on the intersection of the signature genes, yielding differentially expressed genes (DEGs).

The LASSO regression model was established using the package “glmnet” (4.1.7), and the RF model was constructed using the package “randomForest” (4.7.1.1), with “mtry” and “ntree” in RF set to 3 and 500, respectively [23]. DEGs were obtained using package “limma,” the adjusted *p*-value < 0.05 was considered statistically significant.

### Identification and further analysis of DEGs

We utilized the “dplyr,” “tidyr,” and “CMplot” packages to construct Manhattan plot for visualizing the chromosomal locations of DEGs. We utilized the STRING database [24] (http://string-db.org, version 12.0) to identify potential targets, selecting genes with scores exceeding 0.7 for protein–protein interaction (PPI) data and image acquisition. Subsequently, we used Cytoscape (V3.10.1) to further analyze gene correlation [25]. We obtained gene copy number files for CRC patients from UCSC Xena (UCSC Xena (http://xenabrowser.net)) and visualized the copy number variation frequency of DEGs.

The Gene Ontology (GO) and Kyoto Encyclopedia of Genes and Genomes (KEGG) enrichment analyses on the DEGs were executed through the implementation of the “ClusterProfiler” package. The p-value < 0.05 was considered statistically significant. Moreover, to control for false positives, a q-value filter of 0.05 was applied, ensuring robustness in the face of multiple testing. Visualization of the GO and KEGG outcomes was effectuated employing the “ggplot2” package.

### Subtypes construction based on DEGs and immune infiltration analysis

The process of consensus clustering entails the discernment of molecularly related subtypes through the application of the Consensus Cluster Plus algorithm [26]. Classification of samples is achieved by the determination of a consensus matrix, where the cluster number (*k* value) ranges from 2–9. The optimal *k* value, established at 2, is deduced through the identification of the approximate maximum value of the cumulative distribution function indicator. The validity of this determination is subsequently verified through the execution of principal component analysis (PCA) on the mRNA expression profiles.

The transformation of the normalized gene expression matrix into a relative abundance matrix of 22 distinct immune cell types was achieved through the application of the CIBERSORT algorithm [27]. From the initial 505 samples, we retained 404 samples for further analysis, adhering to the criterion of *p*-value < 0.05. Based on the typing results, we extracted the immune cell infiltration matrix of CRLM samples. The “reshape2” and “ggpubr” packages were used to visualize the different infiltration between subtypes.

### Construction and validation of nomogram model for predicting the occurrence of CRLM

The 505 samples from GSE81582, GSE73255, GSE41258, and GSE49355 served as training data, while the 189 samples from GSE14095 were used for external validation. Based on the DEGs, we constructed a nomogram model, using the packages “rms” (6.7.0) and “rmda” (1.6). Calibration plots and decision curve analysis (DCA) were employed to validate the model’s feasibility, while the receiver operating characteristic (ROC) curve was used to validate its accuracy.

### Development nomogram for prognostic assessment in CRLM patients

A cohort comprising 133 patients with synchronous liver metastases and survival information was extracted from the GSE15926 dataset. The DEGs previously identified for CRLM patients, obtained through the methods described in Sect. “Data processing and NETRGs expression levels acquisition” and “Establishment of LASSO, RF and acquisition of DEGs,” were integrated with the GSE15926 dataset using the “limma” package for further analyses.

For additional modeling and analysis, the data underwent 1000 rounds of grouping (*n* = 1000), and through iterative loops (for (i in 1:n)), generated new training sets and test sets in each iteration. During iteration, we applied the Lasso regression model for feature selection and model construction.

To assess the impact of these DEGs on patient survival, the “survival” and “survminer” packages in R were utilized. This involved the execution of survival analysis, including the generation of Kaplan–Meier survival curves and log-rank tests. DEGs significantly associated with patient survival were identified, and their significance was rigorously determined based on p-values.

Subsequently, a nomogram model for prognostic assessment was meticulously constructed using the packages “rms” and “rmda” packages. This predictive nomogram integrated the identified DEGs associated with survival outcomes. To validate the reliability of the nomogram, internal validation was performed, and the performance of the nomogram was further evaluated through the generation of receiver ROC curves for the internally validated model.

### Comprehensive analysis of CYP4F3

We reclassified CRLM samples based on the median expression level of CYP4F3 and used the “limma” package to analyze and visualize differential gene expression (DGE) between the two groups. From the DGE, we constructed PPI and miRNA-mRNA network models. We procured input files for the miRNA regulatory network and delved into the upstream regulation of CYP4F3.

To discern the differences in biological processes and potential mechanisms between the groups, we conducted GO, KEGG, and Gene Set Enrichment Analysis (GSEA) on the RNA-seq dataset of CRLM. A dual criterion of *p*-value < 0.05 and *q*-value < 0.05 was employed to identify statistically significant results. Based on the immune cell infiltration matrix of CRLM, we used the packages “reshape2” and “ggpubr” to visualize the differences.

### Tissues acquisition and immunohistochemistry staining

We collected normal peritumoral tissue, CRC tissue, and CRLM tissue from 80 patients with CRLM through surgical resection. The diagnoses were confirmed based on histopathological examination of the tissue samples. Tumor specimens were fixed in paraformaldehyde solution for 24 h. Paraffin Sects. (4 μm thick) were deparaffinized, rehydrated, subjected to antigen retrieval, and then incubated with the corresponding primary antibodies at 4 °C overnight. The immunohistochemistry (IHC) assays were developed using a DAB solution for the chromogenic reaction. The primary antibody used was anti-CYP4F3 (rabbit, bs-14160R; Bioss), using Image J and GraphPad Prism 9 for semi-quantitative analysis of fluorescence staining intensity. This study received approval from the Ethics Committee of the Sixth People’s Hospital affiliated to Shanghai Jiao Tong University School of Medicine.

### Cell culture, transient transfection, and validation

Mouse colon carcinoma cell line CT26 was obtained from BNCC (Beijing, China). CT26 cells were cultured in DMEM F12 with 10% FBS (Gibco, Thermo Fisher, USA). Cells were grown at 37 °C in a humidified environment containing 5% CO2. The target sequences for CYP4F3 siRNA are shown in Supplementary Table 1.

Using the TRIzol reagent (Sigma-Aldrich, USA), total RNA was extracted from cell lines. Then, using FastStart Universal SYBR® Green Master (Roche, USA) and a Roche LightCycler 480 PCR System, a quantitative reverse transcription-polymerase chain reaction was carried out utilizing the RNA from each sample (2 ug) (Roche, USA). In a 20 ul reaction volume, the cDNA was utilized as a template along with 10 ul of PCR mixture, 0.5 ul of forward and reverse primers, 2 ul of cDNA template, and the necessary amount of water. PCR reactions were performed as follows: cycling conditions started with an initial DNA denaturation step at 95 °C for 30 s, followed by 45 cycles at 94 °C for 15 s, at 56 °C for 30 s, and at 72 °C for 20 s. Threshold cycle (CT) readings were collected and normalized to glyceraldehyde 3-phosphate dehydrogenase (GAPDH) levels in all samples using the 2-ΔΔCT method. The mRNA expression levels were compared to normal tissue controls. The sequences of primer pairs for the target genes are shown in Supplementary Table 1.

#### Transwell assay

Transwell assays for migration and invasion of CT26 cell lines were performed. Briefly, cells (5 × 10^4^) were inoculated into chambers coated (for invasion) or uncoated with Matrigel (BD Biosciences, USA) (for migration). Serum-free medium was added to the upper layer and a complete DMEM medium was added to the lower layer. After 24 h of incubation, migrating or invading cells were fixed with 4% paraformaldehyde and stained with 0.1% crystalline violet.

#### Statistical analysis

The entire spectrum of data processing and analyses was executed using *R* software (version 4.3.0). For normally distributed continuous variables, independent student t-tests were employed to assess statistical significance, whereas the Mann–Whitney U test (Wilcoxon rank-sum test) was utilized for non-normally distributed continuous variables. Comparative analysis of statistical significance between two groups of categorical variables was conducted using the Chi-square test. The correlation between immune cells was analyzed using the Spearman test. All statistical *p*-values were two-sided, with *p*-value < 0.05 considered statistically significant.

## Results

### LASSO, RF, and acquisition of DEGs

**Fig. 2:**
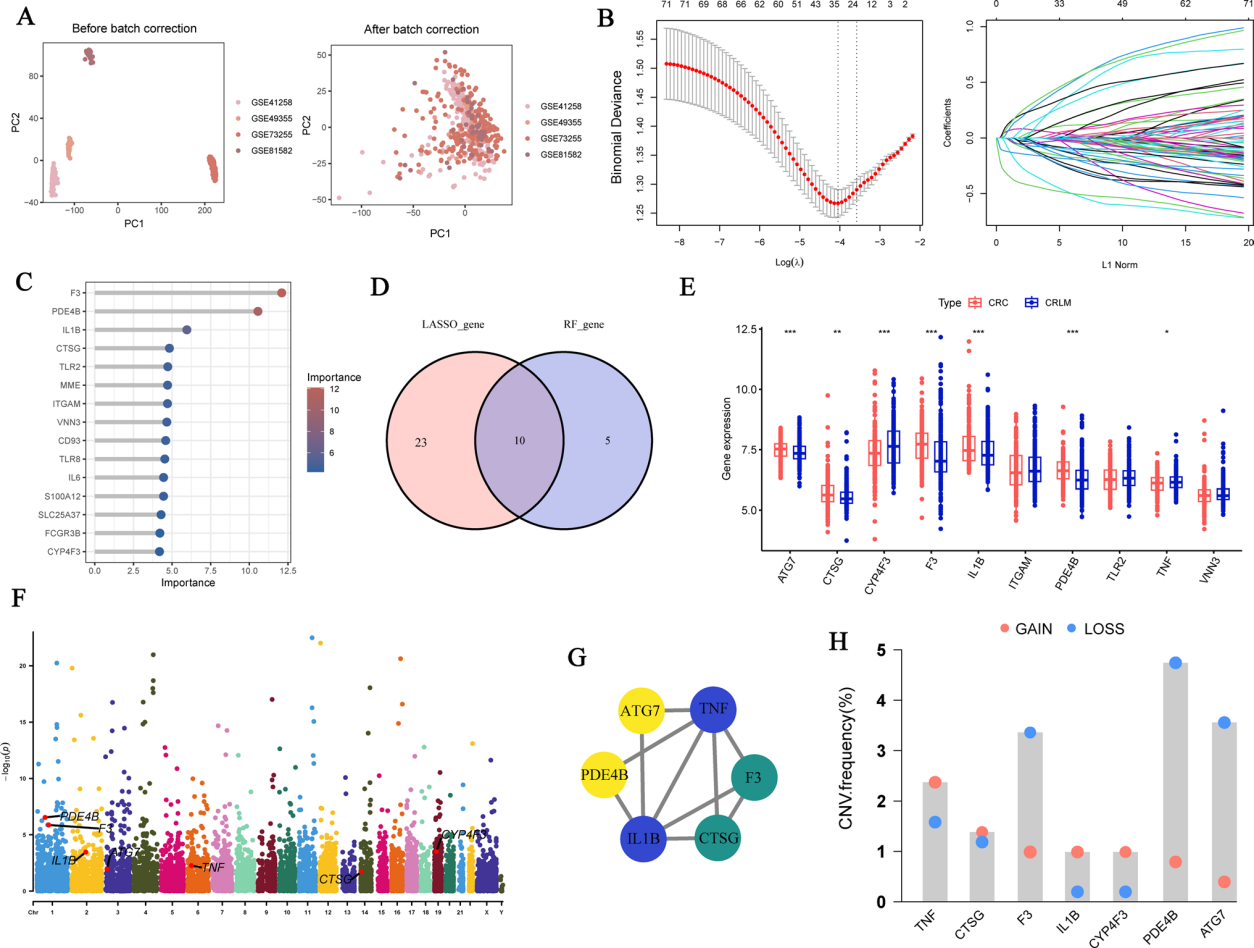
Identification of NETRGs related to CRLM. **A** PCA analysis based on data processing. **B** The LASSO coefficient profiles. **C** Random forest: feature genes with importance scores greater than 2. **D **Ten common genes between the LASSO and RF models. **E** The differential expression boxplot of 10 NETRGs between CRC and CRLM. **F** The chromosomal position of the NETRGs. **G** Interactions between DEGs. **H** Copy number variation frequency in CRC patients. **I** GO&KEGG enrichment analyses of DEGs. (*p < 0.05, **p < 0.01, ***p < 0.001)

We extracted 87 NETRGs through literature reviews (Supplementary Table 2), acquiring their expression levels in four RNA-seq datasets, which were subsequently merged. Batch effects were removed post-merging, and the differences before and after batch effect removal were illustrated using PCA (Fig. 2A**)**. From this, we established a LASSO model and identified 33 NETRGs associated with CRLM (Fig. 2B). Additionally, we constructed a RF model, a supervised machine learning algorithm based on decision trees, capable of handling complex interactions and nonlinear relationships between variables. In the RF model, we displayed the importance scores of the top-ranked genes and gained 15 feature genes with importance scores greater than 2 (Fig. 2C). Cross-validation of the LASSO model and the RF model yielded 10 NETRGs (Fig. 2D). We analyzed the correlation between NETRGs and immune cells, and found that ITGAM has a strong positive correlation with macrophages M2 (Supplementary Fig. 1A).

After performing differential analysis on these genes, we obtained 7 DEGs (ATG7, CTSG, CYP4F3, F3, IL1B, PDE4B, and TNF) (Fig. 2E). Among these, CYP4F3 and TNF were overexpressed in the CRLM tissue, while ATG7, CTSG, F3, IL1B, and PDE4B were found to be opposite, suggesting a potential role for CYP4F3 and TNF in promoting liver metastasis in CRC. Notably, CYP4F3 exhibiting stronger significance than TNF (*p*-value < 0.001), indicating its potential importance in this process.

### Comprehensive analysis of DEGs

The chromosomal locations of these seven genes were found to be dispersed (Fig. 2F). PPI network diagrams revealed interactions among six genes, with CYP4F3 isolated, highlighting its uniqueness (Fig. 2G). We also explored the copy number variation frequency of these genes. F3, PDE4B, and ATG7 had a noticeably higher frequency of copy number gain than loss, while CYP4F3, IL1B, and TNF showed the opposite trend (Fig. 2H).

To further explore whether DEGs are associated with tumor development, we conducted GO and KEGG enrichment analyses. The results showed that the cellular response to lipopolysaccharides ranked high in the biological process (BP) category. KEGG pathway analysis revealed that the AGE-RAGE signaling pathway in diabetic complications and amoebiasis were the top two pathways (Fig. 2I).

### Identification of two subtypes and further analysis

**Fig. 3:**
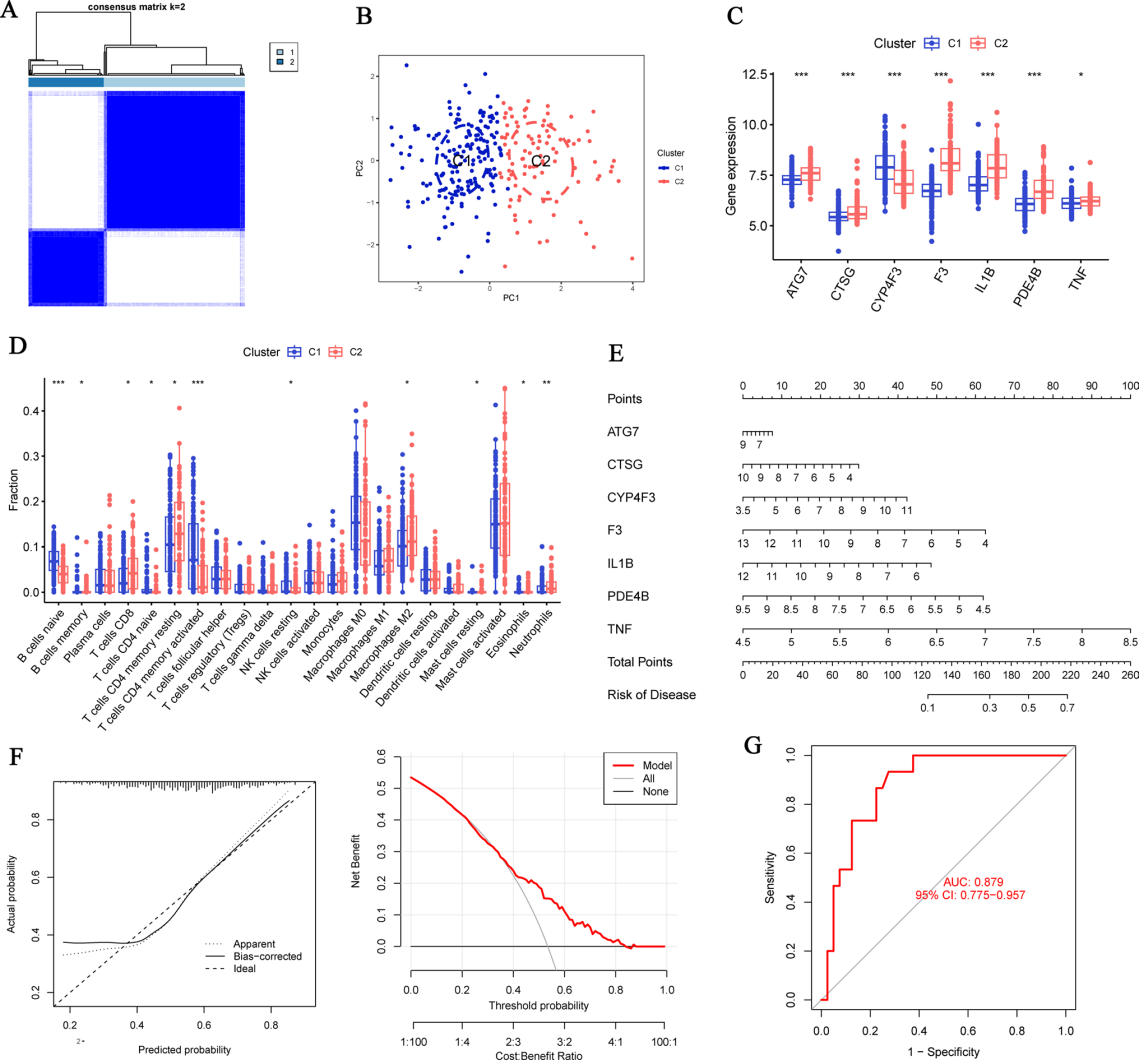
Classification of subtypes and construction of nomogram model. **A** Consensus matrices display the clustering of the seven significant NETRGs for *k* value of 2. **B** PCA illustrates the distinct expression patterns of NETRGs subtypes. **C** The differential expression boxplot of 7 NETRGs between Cluster 1 and Cluster 2. **D** The differential expression boxplot of 22 immune cells between Cluster 1 and Cluster 2. **E** The nomogram model was established according to the seven NETRGs. The incidence was predicted according to total points. A total of 120 points indicated an incidence of 10%, while 220 points indicated an incidence of 70%. **F** The solid and dotted lines are close in the calibration curves in the later stage, the red line representing the NETRGs in the decision curve deviating from the gray and black lines also proved the feasibility of the model. **G** The ROC curves showed the nomogram model with a 0.879 predictive value. (*p < 0.05, **p < 0.01, ***p < 0.001)

We conducted consensus clustering based on 7 DEGs to categorize the CRLM samples into two subtypes (Cluster 1, Cluster 2) (Fig. 3A). We also identified the category through PCA, demonstrating that these 7 genes could effectively differentiate the two CRLM subtypes (Fig. 3B). To explore the biological differences between the two clusters, we generated boxplots (Fig. 3C). We noted that, with the exception of CYP4F3, which was significantly overexpressed in Cluster 1 (*p* < 0.001), the other genes were more highly expressed in Cluster 2. We infer that Cluster 1 subtype exhibits a higher degree of invasiveness. CYP4F3, among these 7 DEGs, appears to be distinctive and positively correlated with the occurrence of CRLM, potentially serving as a key factor in distinguishing liver metastasis.

We also examined the differences in immune cell infiltration among the clusters (Fig. 3D). Compared to Cluster 2, Cluster 1 had a higher number of naïve B cells and T cells CD4 memory activated (*p* < 0.001), while the level of neutrophil infiltration was lower (*p* < 0.01).

### Construction and validation of nomogram

We developed a nomogram model using the 7 DEGs (Fig. 3E). In this model, a total score predicting the occurrence rate of CRLM was calculated by individually scoring each gene. The solid and dashed lines in the calibration curve deviate significantly from each other in the early stage but gradually converge later, indicating that the model’s early fit to the data is not sufficient, resulting in lower prediction accuracy, while the red line representing NETs genes in the decision curve deviated from the gray and black lines in the later stage (Fig. 3F). These may be due to the insufficient representativeness of the training set data. GSE14095 was used for external validation. The ROC curve showed that the AUC value of the validation set was 0.879, indicating that the nomogram model had good discrimination ability (Fig. 3G). The above results all indicate that this model has good potential in predicting liver metastasis in CRC patients. However, we also acknowledge the need for further validation with larger and more diverse data to confirm its predictive effect.

### Comparing prognostic significance of DEGs in CRC and CRLM and constructing a prognostic nomogram for CRLM

We examined the impact of DEGs on the prognosis of CRC patients using supple (GEPIA (Gene Expression Profiling Interactive Analysis) (cancer-pku.cn)) and conducted a univariate COX regression analysis using GSE159216 for comparison (Supplementary Table 3). Our results suggest that CYP4F3, ATG7, and PDE4B do not impact the prognosis of patients with CRC, but play a role in influencing the prognosis of patients with CRLM (Supplementary Fig. 1B). Contrary to our initial inference, an analysis of the GSE159216 dataset indicates that CRLM patients exhibiting overexpression of CYP4F3 demonstrate a more favorable prognosis. To delve into the underlying reasons for this unexpected finding, we conducted an exploration of prognostic data across four stages of CRLM patients using the Kaplan–Meier Plotter platform (https://kmplot.com/analysis/). The findings uncovered that CRLM patients with low-expression levels of CYP4F3 exhibit a more favorable prognosis (Supplementary Fig. 1C). The results for CYP4F3 are consistent with our earlier predictions, showing elevated expression in both CRC and CRLM patients (Supplementary Fig. 1D). While its expression does not impact the prognosis of CRC patients, it significantly influences the prognosis of synchronous liver metastasis patients within our GSE159216 dataset.

**Fig. 4:**
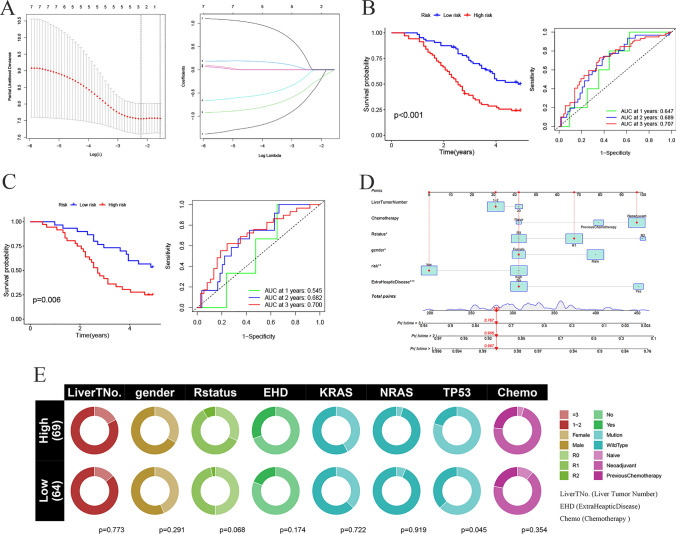
**A** The LASSO coefficient profiles. **B** High- and low-risk group Kaplan–Meier curves and ROC curves. **C** Kaplan–Meier curves and ROC curves for the high- and low-risk groups in the validation set. **D** Prognostic nomogram for CRLM. **E **Clinical information of CRLM patients. (*p < 0.05, **p < 0.01, ***p < 0.001)

Subsequently, we utilized the information derived from the Lasso regression and multivariable Cox modeling to construct a prognostic model for CRLM patients (Fig. 4A). Evidencing substantial survival distinctions (*p* < 0.001), the model effectively discriminated between the high-risk and low-risk groups, as depicted through Kaplan–Meier survival curves and receiver operating characteristic (ROC) curves spanning 1, 2, and 3 years, inclusive of all patients (Fig. 4B). The corresponding ROC values were 0.647, 0.689, and 0.707, respectively. Internal validation of the prognostic model was conducted and revealing notable survival differences between the high-risk and low-risk groups (*p* = 0.006) with ROC values of 0.545, 0.682, and 0.700 for 1, 2, and 3 years, respectively (Fig. 4C).

Encouraged by the observed accuracy of the model, we integrated clinical information to construct a nomogram. In this nomogram, factors such as the number of liver metastases and chemotherapy history did not impact prognosis while extra hepatic diseases emerged as the most influential prognostic factor, followed by the risk factors identified in our model—the genes CYP4F3 and ATG7. Interestingly, gender exhibited some impact on prognosis in our cohort (Fig. 4D), necessitating further validation in larger cohorts. Unfortunately, suitable datasets for in-depth exploration were not identified. Finally, we conducted a comparative analysis of clinical features between the high-risk and low-risk groups, revealing significant differences of TP53 (Fig. 4E). These findings collectively underscore the prognostic utility of the constructed model, emphasizing the pivotal roles of CYP4F3 and ATG7 in CRLM patient outcomes.

### Comprehensive analysis of CYP4F3

**Fig. 5:**
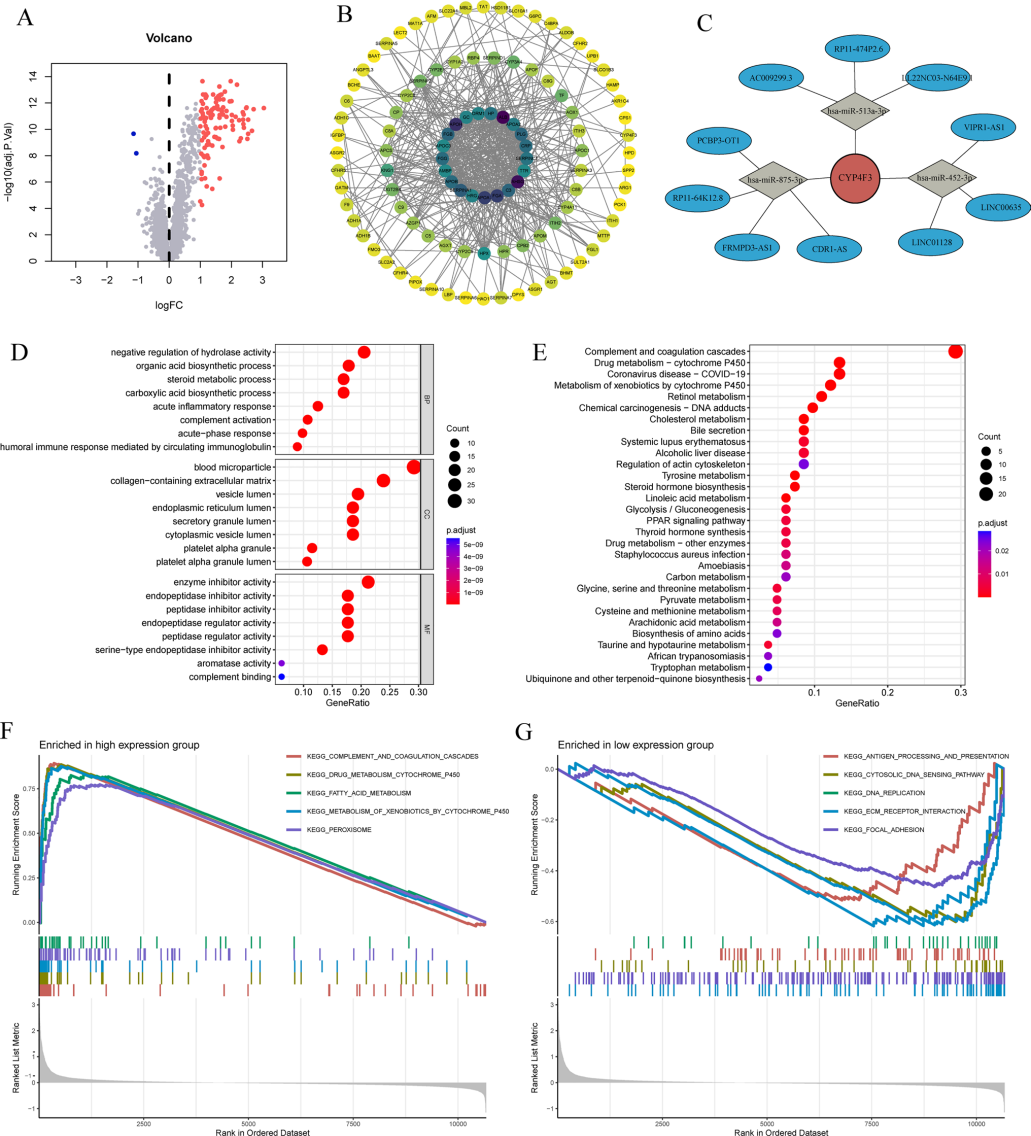
**A** The differential expression volcano plot of genes between high-expression and low-expression group. **B** Interactions between DEGs. **C** The regulatory network of CYP4F3. **D** DEGs KEGG analysis. **E** DEGs GO analysis. **F** GSEA enrichment analysis of DEGs in high-expression group. **G** GSEA enrichment analysis of DEGs in and low-expression group

In the aforementioned study, we deduced that CYP4F3 could be a potential key predictor of CRLM. We calculated the expression levels of CYP4F3 in the samples and, based on the median, reclassified the CRLM patients into two groups: those with expression levels below the median and those with expression levels above the median, and identified DGE (*p* < 0.05 and |log2FC|> 1). The volcano plot revealed that, compared to high-expression group, there were 114 upregulated genes and 2 downregulated genes in low-expression group (Fig. 5A). Moreover, CYP4F3 showed correlation with other upregulated genes (Fig. 5B).

In an effort to comprehend the interactions between CYP4F3 and other genes, we established PPI and gene network models, indicated that CYP4F3 can directly interact with CYP2C8, CYP4A11, CYP2E1, and CYP2C9, and indirectly modulate the expression levels of genes such as CYP3A4, FMO3, and AOX1 (Fig. 5B). Furthermore, we generated a miRNA-mRNA regulatory network diagram, which unveils that CYP4F3 is interacting with has-miR-875-3p, has-miR-513a-3p, and has-miR-452-3p (Fig. 5C).

### Further analysis of DGE between high-expression and low-expression group

To explore the functional differences among the DGE between high-expression and low-expression group, we conducted GO and KEGG analyses, and visualized the results. GO analysis revealed that the negative regulation of hydrolase activity and organic acid biosynthesis process ranked the top two in the BP category, while blood microparticle and enzyme inhibitor activity were the top-ranked in the cellular component (CC) and molecular function (MF) categories, respectively (Fig. 5D). KEGG analysis showed that among the pathways enriched by these differential mRNAs, the complement and coagulation cascades, drug metabolism—cytochrome P450 and coronavirus disease COVID-19 were the top three pathways (Fig. 5E).

To address potential biases in enrichment analysis of intersection genes, we used the “c2.cp.kegg.Hs.symbols.gmt” gene set to perform GSEA on the CRLM samples. Consistent with the KEGG analysis results, in high-expression group, the gene set was enriched in the upregulated pathways of complement and coagulation cascades, drug metabolism cytochrome P450 and metabolism of xenobiotic by cytochrome P450 (Fig. 5F). In low-expression group, the gene set was enriched in the downregulated pathways of antigen processing and presentation and cytosolic DNA sensing pathway (Fig. 5G).

**Fig. 6:**
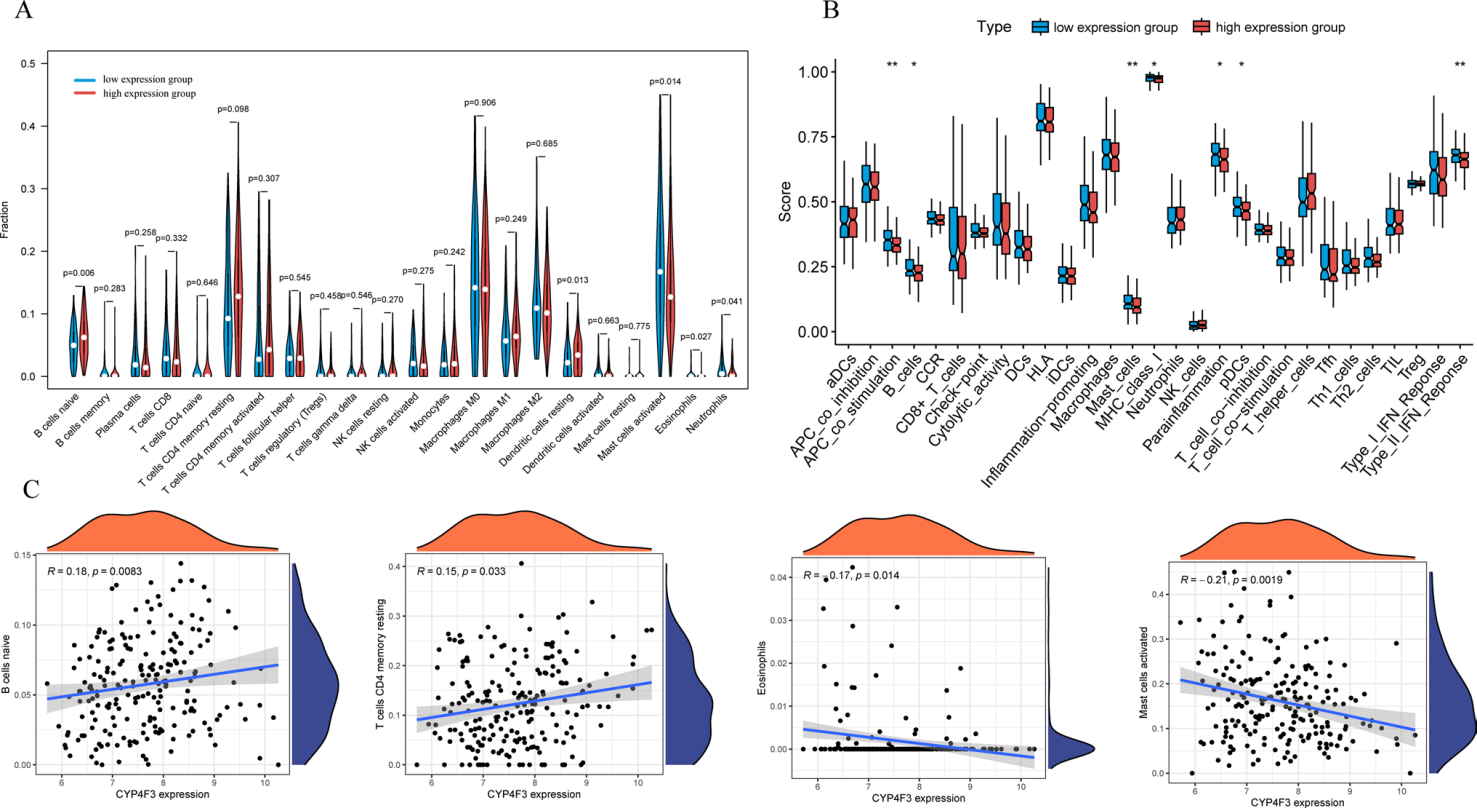
**A** Immune infiltration between high-expression and low-expression group. **B** Immune checkpoints scores between two groups. **C** Correlation among CYP4F3 and immune cells

We further conducted an investigation into the impact of CYP4F3 expression levels on immune levels. We validated the differences in immune infiltration between high-expression and low-expression groups, and found that compared to low-expression group, high-expression group had relatively fewer mast cells activated, eosinophils, and neutrophils, but more infiltration of B cells naïve and dendritic cells resting (Fig. 6A). Further analysis revealed that in the immune functions of high-expression group samples, the scores of APC co-stimulation, Mast cells, and Type II IFN Response were relatively lower (Fig. 6B). Additionally, the expression level of CYP4F3 was negatively correlated with mast cells activated and eosinophils, and positively correlated with B cells naïve and T cells CD4 memory resting (Spearman’s coefficient) (Fig. 6C).

### Expression and invasion validation of CYP4F3

**Fig. 7:**
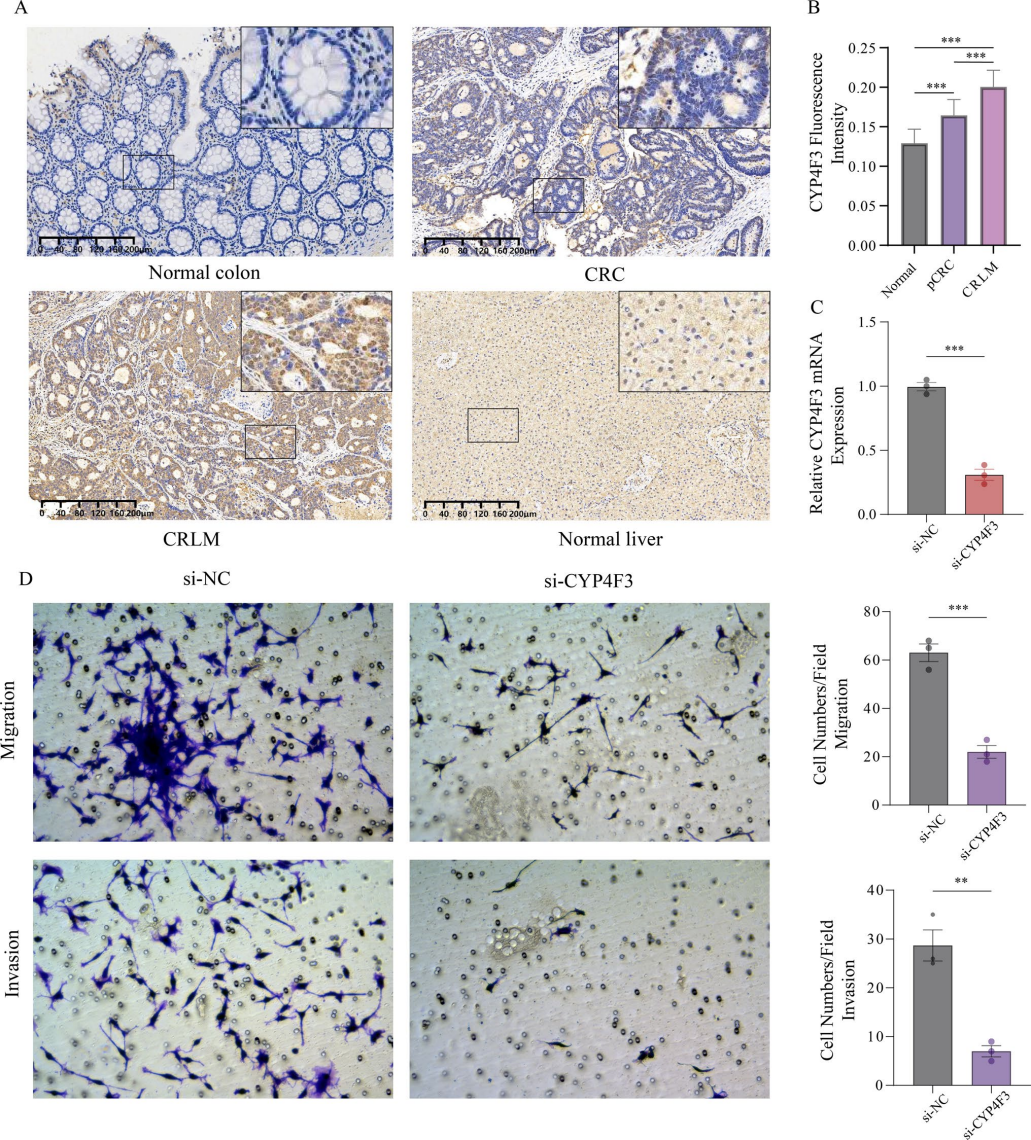
**A** Immunohistochemistry of CYP4F3 in normal colon tissue, primary colorectal cancer tissue, liver metastasis tissues and normal liver tissue from CRLM patients. **B** Semi-quantitative analysis of immunofluorescence staining intensity. A statistically significant difference between normal tissues, CRC tissues and CRLM tissues, with a *p*-value < 0.001. **C** Relative mRNA expression of CYP4F3 in si-NC group and si-CYP4F3 group. **D** Transwell assay in CT26 cells after transfection of si-NC and si-CYP4F3

The protein expression level of CYP4F3 was confirmed through IHC. Generally, normal intestinal tissues exhibited lower protein expression levels compared to CRC tissues, while CRLM tissues displayed the highest protein expression levels, higher than normal liver tissues (Fig. 7A). By semi-quantitative analysis of tissue fluorescence staining intensity, significant differences in expression levels were observed between normal tissues, CRC tissues and CRLM tissues (Fig. 7B).

### Functional validation of CYP4F3 in vitro

CT26 cells were cultured in vitro and subsequently divided into two groups based on transfection plasmid: si-NC and si-CYP4F3. The mRNA expression levels of CYP4F3 in the transfected cell lines were assessed using RT-qPCR (Fig. 7C). Furthermore, we conducted Transwell invasion and migration assays. Results of experiments revealed a significantly lower number of CRC cells passing through the Transwell chamber in the si-CYP4F3 group compared to the si-NC group (Fig. 7D), indicating that CYP4F3 downregulation suppressed the invasion and migration abilities of CT26 cells.

## Discussions

CRC is a frequent clinical diagnosis, with prognosis significantly impacted by metastasis. The liver, largely due to its portal vein system blood supply, is the primary site of metastasis [28]. Despite the range of treatment options for CRLM, including surgery, ablation therapy, chemotherapy, anti-angiogenic therapy, and immunotherapy, many patients still experience metachronous liver metastasis [29, 30]. Consequently, early detection and treatment of CRLM are vital for improving prognosis.

Indeed, studies have been made in developing models to predict CRLM. For example, Li et al. developed an imaging genomics paradigm and individualized nomogram model for predicting primary tumor CRLM using enhanced computed tomography (CT) images from 50 patients [31]. Studies have explored the prognostic aspects of CRLM, with the consistent finding indicating the influence of gender on prognosis [32, 33]. Our study distinguishes itself from prior research by adopting a novel perspective centered around NETRGs. We devised two predictive models, introduced an innovative classification method, and investigated the involvement of CYP4F3 in the context of CRLM.

Recent studies have highlighted the crucial role of NETs formation in the development of colorectal cancer [34]. The viability of employing NETRGs to forecast the onset and prognosis of CRLM has been substantiated. In this investigation, we discerned seven DEGs when comparing CRC and CRLM samples, followed by enrichment analysis. GO analysis revealed that DEGs participate in the biological processes associated with cellular response to lipopolysaccharides, which are linked to inflammatory responses. Advanced glycation end products (AGEs) are primarily associated with chronic hyperglycemia; however, emerging evidence suggests their involvement in activating multiple signaling pathways related to cell survival, inflammation, and cancer progression [35]. These findings underscore the importance of DEGs in tumor biology, particularly in modulating inflammatory responses. Subsequently, we scrutinize their correlation with immune cells. A robust positive correlation was unveiled between ITGAM and M2 macrophages, intimating that ITGAM potentially assumes a pivotal role in modulating the differentiation or functionality of M2 macrophages. This modulation, in turn, is posited to impact pertinent immune and inflammatory responses and, consequently, the incidence of liver metastasis.

We utilized seven DEGs for the categorization of CRLM patients into distinct subtypes, denoted as Cluster 1 and Cluster 2. Subsequently, we discerned variations in immune infiltration patterns between these identified subtypes. Our inference posits that the comparatively diminished immune level within Cluster 1, as evidenced by a heightened proportion of resting B cells, activated CD4 memory T cells, and reduced levels of central memory cell infiltration, may contribute to a diminished capability to eradicate tumor cells. This scenario heightens the likelihood of immune escape, consequently making Cluster 1 more aggressive. The elevated expression of CYP4F3 in Cluster 1 implies a positive correlation between CYP4F3 and higher invasiveness.

In the stratification of CRLM patients according to CYP4F3 expression, we observed an upregulation in pathways associated with complement and coagulation cascades, drug metabolism cytochrome P450, fatty acid metabolism, peroxisome, and metabolism of xenobiotics by cytochrome P450 within the high-expression group. The first pathway encompasses a series of biochemical reactions crucial for both immune response and hemostasis in the body, with significant implications in the tumor microenvironment, potentially contributing to tumor invasion and metastasis. Cytochrome P450 enzymes catalyze oxidative reactions, while peroxisomes are involved in fatty acid oxidation. Cellular regulation of redox reactions is intricate, as they can either promote cell proliferation and survival or inhibit growth and induce cell death. Cancer cells may adapt to oxidative stress environments by enhancing antioxidant defenses, influencing metabolic pathways, and reinforcing survival signaling. Consequently, an increase in redox reactions may facilitate cancer cell proliferation and metastasis. The upregulation of the latter four pathways suggests that CYP4F3 may influence pathways associated with oxidative and metabolic reactions, thereby impacting cancer invasion and metastasis.

The CYP superfamily, comprising heme-containing proteins, plays a crucial role in fatty acid metabolism, cholesterol production, and steroid hormone synthesis [36]. Its subfamily member, CYP4F3, undergoes selective splicing to produce CYP4F3A and CYP4F3B [37]. CYP4F3A, primarily expressed in leukocytes, can inactivate leukotriene B4 (LTB4), which is a chemotactic agent for neutrophils and considered as an initiator of immune responses [38, 39]. Conversely, CYP4F3B is predominantly expressed in the liver, preferentially converting arachidonic acid into 20-HETE [37]. Given that leukotrienes and arachidonic acid are mature inflammatory mediators, CYP4F3 plays a significant role in the inactivation and degradation of these mediators. This aligns with our GO and KEGG enrichment results, suggesting that CYP4F3 influences DEGs, and inhibits a series of enzymatic reactions, and thereby impacts the inflammatory response. However, CYP4F3’s role in the inflammatory response may be bidirectional, both promoting and inhibiting, potentially depending on varying physiological environments and pathological conditions. As Ni et al. have demonstrated, metabolites mediated by CYP ω-hydroxylase exhibit pleiotropic effects in numerous inflammation-related diseases [40].

Studies have indeed associated CYP4F3 with cancer [41, 42]. The downstream effects of CYP4 in lung and kidney cancers are reportedly mediated by PI3K/Akt, ERK1/2, and small GTPase Ras [43, 44]. Its metabolic product, 20-HETE, is also known to play a pivotal role in cell growth and cancer development. Cárdenas et al. demonstrated that the 20-HETE-GPR75 receptor is implicated in activating intracellular signaling stimulated in cellular malignant transformation, thereby increasing the invasiveness of prostate cancer cells [45]. Inhibition of 20-HETE synthesis has been shown to reduce the migration and invasion of metastatic triple-negative breast cancer cell lines, as well as decrease primary tumor growth and lung metastasis in vivo [46]. However, the specific mechanisms of CYP4F3 in cancer onset, progression, and metastasis remain largely elusive.

We devised a prognostic model for patients with CRLM, and internal validation affirmed its robust predictive efficacy. Expanding on this model, we incorporated clinical data to formulate a diagnostic nomogram. This nomogram delineates the prognostic significance of risk factors, extra hepatic diseases, and other variables for CRLM patients, providing valuable insights for clinicians to evaluate patient prognosis and tailor personalized treatment strategies.

In developing a prognostic assessment model for CRLM using the GSE159216 dataset, we uncovered a compelling observation. Within this dataset, CYP4F3 significantly impacts the prognosis of patients with synchronous liver metastases. Contrary to our initial predictions, CRLM patients with CYP4F3 overexpression manifest a more favorable prognosis. We posit that this phenomenon may be linked to patients in this dataset undergoing adjuvant therapy before surgery. Enzymes of the CYP family play a pivotal role in drug metabolism within the human body. Research suggests that chemotherapy drugs can stimulate enhanced transcription of CYP family genes in liver cells, thereby augmenting the systemic clearance of drugs, primarily through the action of CYP3A [47]. Consequently, we deduce that the overexpression of CYP4F3 may influence the expression of CYP3A or drug clearance function. However, the specific interactions, regulatory mechanisms, and their relevance in the human body are currently understood to be relatively limited. Evidence also indicates that CYP4F3 directly impacts the metabolism of imatinib [48], suggesting that CYP4F3 overexpression in this dataset may attenuate the metabolism of adjuvant therapy drugs, thereby enhancing their efficacy. This underscores the intricate nature of prognostic factors in CRLM, emphasizing the imperative for further investigation. Nonetheless, this introduces a degree of uncertainty regarding the model’s accuracy, necessitating validation in larger cohorts.

To further explore the biological functions linked to CYP4F3 expression levels, we examined its correlation with immune levels. In line with previous studies, we observed differences in immune levels between samples with varying CYP4F3 expression levels. Samples with high CYP4F3 expression levels exhibited fewer activated mast cells, eosinophils, and neutrophils, but more naive B cells and resting dendritic cells. Scores for APC co-stimulation, Mast cells, and Type II IFN Response were relatively lower in these samples. These findings further underscore CYP4F3’s critical role in modulating inflammatory responses, potentially suppressing immune levels, facilitating immune escape, and enhancing CRC progression, invasiveness, and metastasis.

To investigate upstream regulatory relationships further, we utilized STRING and Cytoscape to predict miRNAs interacting with CYP4F3. Our analysis revealed that CYP4F3 interacts with three miRNAs: has-miR-875-3p, has-miR-513a-3p, and has-miR-452-3p. miRNAs can inhibit the translation of their target genes by binding to their messenger RNA 3’-untranslated regions, a post-transcriptional regulation that occurs in pathophysiological processes, including CRC [47–49]. Notably, it has been reported that has-miR-513a-3p and its target genes influence the proliferation and glycolysis of CRC cells [50]. These findings align with the data we have mined, further emphasizing the potential role of these miRNAs in CRC progression.

However, we acknowledge several limitations. Firstly, due to the scarcity of large datasets meeting our criteria in the GEO database, we amalgamated four chips from different platforms, leading to batch effects. Secondly, the multidimensional nature of CRLM’s occurrence mechanism means our discussion from the perspective of inflammatory response and immune infiltration may not be exhaustive. Thirdly, while we have demonstrated CYP4F3 expression through IHC, further in vivo and in vitro experiments are imperative for a comprehensive elucidation of the molecular mechanisms underlying the gene’s clinical applicability. In addition, compared to bulk RNA-seq, scRNA-seq analysis could offer more valuable insights into immune cell heterogeneity. We plan to conduct scRNA-seq analysis in future research for further exploration.

## Supplementary Information

Below is the link to the electronic supplementary material.

Supplementary file1 (TIF 56937 KB)

Supplementary file2 (XLSX 33 KB)

Supplementary file3 (DOCX 15 KB)

Supplementary file4 (XLSX 15 KB)

Supplementary file5 (XLSX 593 KB)

## Data Availability

The RNA-seq data for our study were retrieved from the Gene Expression Omnibus (GEO) database, accessible at https://www.ncbi.nlm.nih.gov/geo/. The datasets with accession numbers GSE81582, GSE73255, GSE41258, GSE49355, GSE14095, and GSE159216 were utilized in our analysis. Researchers interested in accessing and further exploring the raw RNA-seq data can find it in the specified GEO accession numbers.

## Abbreviations

CRC: Colorectal cancer
CRLM: Colorectal cancer liver metastasis
NETs: Neutrophil extracellular traps
DDR1: Discoid domain receptor 1
NETRGs: NET-related genes
GEO: Gene expression omnibus
DEGs: Differentially expressed genes
DGE: Differential gene expression
LASSO: Least absolute shrinkage and selection operator
RF: Random forest
CYP: Cytochrome P450
PDCA: Pancreatic ductal adenocarcinoma
GSEA: Gene set enrichment analysis
GO: Gene ontology
KEGG: Kyoto Encyclopedia of genes and genomes
RNA-SEQ: RNA-Sequencing
pCRC: Primary colorectal cancer
PCA: Principal component analysis
DCA: Decision curve analysis
ROC: Receiver operating characteristic
IHC: Immunohistochemistry
BP: Biological process
PPI: Protein–protein interaction
GEPIA: Gene expression profiling interactive analysis
CC: Cellular component
MF: Molecular function
NLR: Neutrophil-to-lymphocyte ratio
LDH: Lactate dehydrogenase
LTB4: Leukotriene B4
HETE: Hydroxyeicosatetraenoic acid
