# Supplementary figures and images for: Establishment of a neutrophil extracellular trap-related prognostic signature for colorectal cancer liver metastasis and expression validation of CYP4F3
Source: Clin Exp Med. 2024 May 25;24(1):112. doi: 10.1007/s10238-024-01378-0 (PMC11127854; doi:10.1007/s10238-024-01378-0)

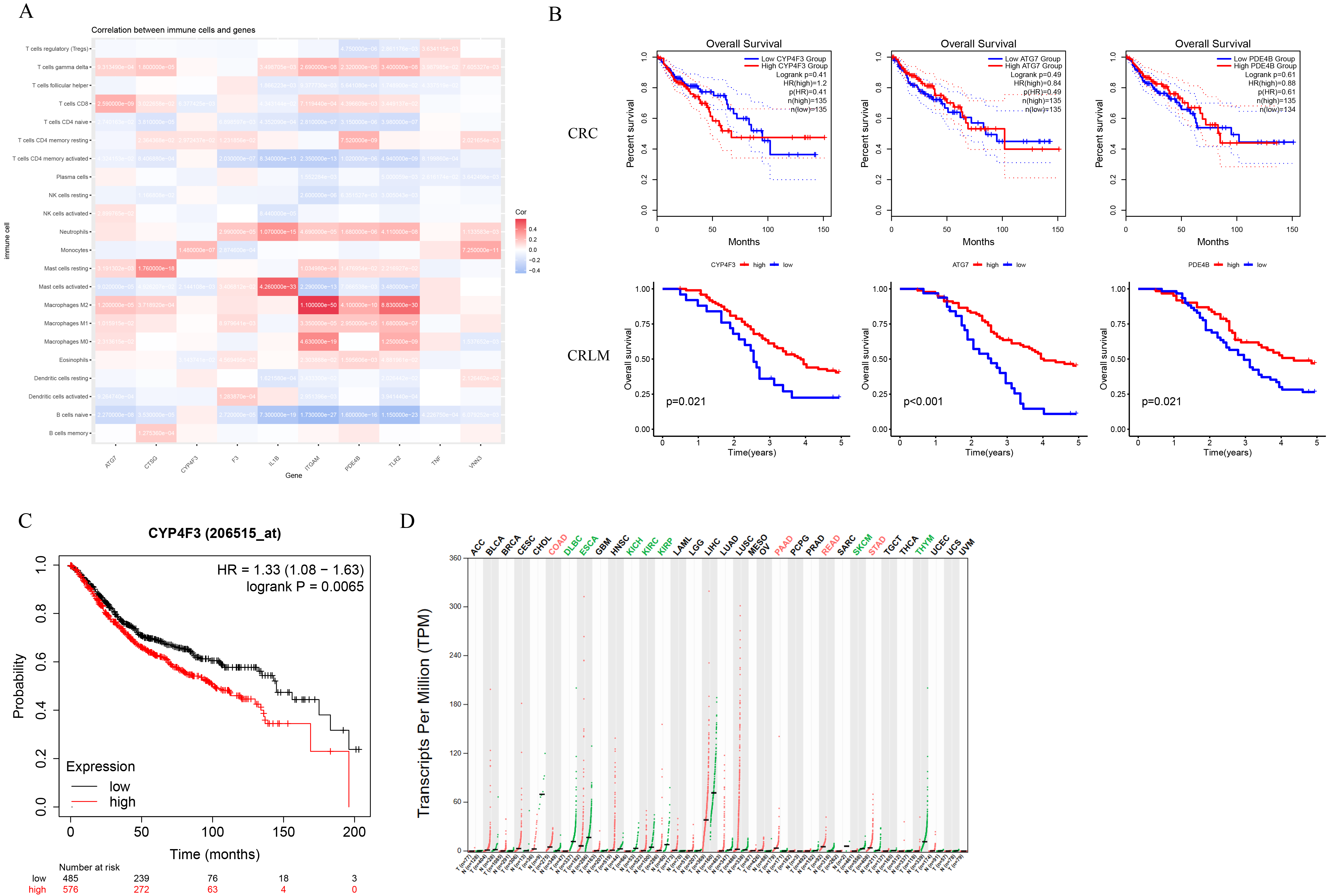

Supplement: Supplementary file 1 — Supplementary file1 (TIF 56937 KB) [file 10238_2024_1378_MOESM1_ESM.tif]
