# Supplementary material for: Establishment of a neutrophil extracellular trap-related prognostic signature for colorectal cancer liver metastasis and expression validation of CYP4F3
Source: Clin Exp Med. 2024 May 25;24(1):112. doi: 10.1007/s10238-024-01378-0 (PMC11127854; doi:10.1007/s10238-024-01378-0)
Supplement: Supplementary file 3 — Supplementary file3 (DOCX 15 KB) [file 10238_2024_1378_MOESM3_ESM.docx]

The target sequences for CYP4F3 siRNA:

| Gene | target sequence (5’-3’) |
| --- | --- |
| si-CYP4F3 | AGGGGAGAGACAGACCTGGGAAA |

The sequences of primer pairs for the target genes:

| Gene | Forward primer sequence（5’-3’） | Reverse primer sequence（5’-3’） |
| --- | --- | --- |
| CYP4F3 | GGAAAGGCTCTGTCTGATGAGG | TGGGTGTCTTGCCAGGTTGTAC |
| GAPDH | CATCACTGCCACCCAGAAGACTG | ATGCCAGTGAGCTTCCCGTTCAG |
